# De-Escalation of Antiplatelet Treatment in Patients with Myocardial Infarction Who Underwent Percutaneous Coronary Intervention: A Review of the Current Literature

**DOI:** 10.3390/jcm9092983

**Published:** 2020-09-15

**Authors:** Daniel MF Claassens, Dirk Sibbing

**Affiliations:** 1 Department of Cardiology, St. Antonius Hospital, 3435CM Nieuwegein, The Netherlands; 2 Privatklinik Lauterbacher Mühle am Ostersee, 82402 Iffeldorf, Germany; Dirk.Sibbing@med.uni-muenchen.de; 3 Department of Cardiology, Klinikum der Universität München, Ludwig-Maximilians-University, 81377 Munich, Germany; 4 DZHK (German Centre for Cardiovascular Research), Partner Site Munich Heart Alliance, 80802 Munich, Germany

**Keywords:** P2Y_12_ inhibitor, clopidogrel, ticagrelor, prasugrel, de-escalation, platelet function testing, genotype-guided, percutaneous coronary intervention, acute coronary syndrome, myocardial infarction

## Abstract

In acute coronary syndrome (ACS) patients undergoing percutaneous coronary intervention (PCI), treatment with the P2Y_12_ inhibitors ticagrelor or prasugrel is recommended over clopidogrel due to a better efficacy, albeit having more bleeding complication. These higher bleeding rates have provoked trials investigating de-escalation from ticagrelor or prasugrel to clopidogrel in the hope of reducing bleeding without increasing thrombotic event rates. In this review, we sought to present an overview of the major trials investigating several different options for de-escalation; unguided, platelet function testing- and genotype-guided. Based on these results, and on other established literature sources, such as guidelines and expert consensus papers, we provide an overview to help decide when and how to de-escalate antiplatelet therapy in ACS patients undergoing PCI.

## 1. Introduction

Patients with myocardial infarction and percutaneous coronary intervention (PCI) require dual antiplatelet therapy (DAPT) consisting of aspirin and a P2Y_12_ inhibitor for at least 6 to 12 months [1,2]. In patients with myocardial infarction, potent platelet inhibition with ticagrelor or prasugrel instead of clopidogrel has been preferred by the major guidelines in the past decade. These recommendations followed after results of the Platelet Inhibition and Patient Outcomes (PLATO) and the Trial to Assess Improvement in Therapeutic Outcomes by Optimizing Platelet Inhibition With Prasugrel Thrombolysis in Myocardial Infarction 38 (TRITON-TIMI 38) trials, demonstrated a reduction in thrombotic events in patients using ticagrelor and prasugrel compared to clopidogrel, and the U.S. Food and Drug Administration (FDA) added a boxed warning regarding the reduced effectiveness of clopidogrel in poor metabolizers [3,4]. However, the increased efficacy of the potent P2Y_12_ inhibitors is hampered by a higher bleeding risk.

Clopidogrel is a prodrug. The active metabolite irreversibly binds to the P2Y_12_ receptor on platelets leading to reduced platelet activation [5]. Patients on clopidogrel demonstrate a wide variability in platelet reactivity and approximately 30% of the patients have an inadequate reduction in platelet reactivity measured using platelet function testing [6]. The most important enzyme in the activation process is encoded by the *CYP2C19* gene. This gene has many different alleles, some of which are considered loss-of-function alleles and can be present in more than 30% of the population [7]. Patients carrying loss-of-function alleles generally show higher residual platelet reactivity and are at an increased risk for thrombotic events [8,9].

## 2. De-Escalation of Antithrombotic Therapy

The higher risk of bleeding in patients on potent P2Y_12_ inhibitors remains present in the chronic treatment phase, while the greatest benefit of the potent drugs are seen early, when the risk of recurrent thrombotic events is highest [10,11]. De-escalation is the process of switching from the potent P2Y_12_ inhibitors prasugrel or ticagrelor to weaker clopidogrel. Despite a lack of evidence supporting de-escalation, de-escalation is common in clinical practice and occurs in up to 30% of patients with myocardial infarction [12,13,14]. This is triggered by both clinical factors (e.g., side effects like dyspnea or (minor) bleedings) and socioeconomic factors (e.g., higher costs of ticagrelor and prasugrel treatment) [12,13,14]. For instance, in the Comparison of Prasugrel and Ticagrelor in the Treatment of Acute Myocardial Infarction (PRAGUE-18) trial, more than one-third of patients de-escalated to clopidogrel for economic reasons [12], while in the POPular AGE trial (including acute coronary syndrome (ACS) patients of 70 years and older), treatment adherence in the ticagrelor and prasugrel group was just 53% during the one year follow-up, mainly due to side effects and a perceived high bleeding risks [13].

This has prompted many observational studies investigating the effects of de-escalation of antiplatelet therapy [15], but in the last few years different randomized controlled trials have been published as well [16,17,18]. These trials investigated several different methods of de-escalation; unguided de-escalation, platelet function testing (PFT)-guided de-escalation and genotype-guided de-escalation. For this review, we focus on randomized controlled trials that investigate de-escalation from ticagrelor or prasugrel to clopidogrel. Results are summarized in Table 1.

## 3. Unguided De-Escalation

The Timing of Platelet Inhibition After Acute Coronary Syndrome (TOPIC) trial investigated unguided de-escalation from a potent platelet inhibitor to clopidogrel 1 month after an ACS [16]. It included 646 patients, 323 randomized to de-escalation of DAPT, and 323 to continuing potent platelet inhibition. It found a significant reduction in bleeding academic research consortium (BARC) 2 and higher bleedings (4.0% vs. 14.9% for de-escalated and potent DAPT, respectively, HR 0.30, 95% confidence interval (CI) 0.18–0.50), with no difference in thrombotic events, consisting of cardiovascular death, unplanned revascularization and stroke (9.3% vs. 11.5%, HR 0.80, 95% CI 0.50–1.29). A pre-specified sub analysis of the TOPIC trial assessed the effect of on-treatment platelet reactivity (on prasugrel or ticagrelor) on clinical outcomes [19]. It found that de-escalation was superior regardless of initial platelet reactivity, but that patients classified as low on-treatment platelet reactivity had the highest risk of experiencing a clinical event (either bleeding or thrombotic) and benefited the most from de-escalation. Though these results seem very promising, several important limitations have to be considered. First, the trial had a small sample size. Second, there was no detailed reporting and external event adjudication on key endpoints and third, a difference in event rates, both thrombotic and bleeding, in favor of de-escalation was already seen prior to actual de-escalation.

The recently published HOST-REDUCE-POLYTECH-ACS was a randomized trial that investigated a different kind of de-escalation. It included 2338 East Asian patients with ACS and PCI who were treated with prasugrel 10mg daily for one month [20]. After one month, patients were randomized to either 5 mg daily for 11 months or continued 10 mg daily for the rest of the year. It found the reduced dose to be superior for a combined bleeding and ischemic outcome composed of all-cause death, myocardial infarction, stent thrombosis, repeat vascularization, stroke, or bleeding academic research consortium (BARC) grade 2 bleeding or higher (7.2% vs. 10.1%, hazard ratio (HR) 0.70, 95% CI 0.52–0.92, *p* = 0.012) [20]. This was driven by a difference in BARC 2 bleeding, with no difference in ischemic outcomes. These results are promising, but there are some important limitations, mostly caused by exclusion of patient groups who cannot be treated by 10mg of prasugrel (history of transient ischemic attack/stroke, Age ≥ 75 years and weight <60 kg). The mean age was only 59 years and due to the weight restriction and the low weight of Korean women, there were very few women (±10%). Furthermore, most ischemic events were repeat revascularization, with all other ischemic endpoints having an incidence of <1%. In addition, the mean body weight was 72 kg, and it is, therefore, unsure if in other populations with much higher mean body weight, such as American and European populations, the same effect is reached. Similar to this trial, a randomized trial investigating de-escalation from a high dose to a low dose ticagrelor (90 mg vs. 60 mg) after 1 week until 1 year is ongoing (NCT04255602).

A meta-analysis from Angiolillo et al. pooled observational studies, which studied de-escalation of treatment from ticagrelor to clopidogrel for various reasons [15]. It found a rate of major adverse cardiac events (MACE) (defined as cardiovascular death, myocardial infarction and stroke) of 2%, a rate of cardiovascular death of 2% and of major bleeding of 1%. Contrary to these results are the results from the Switching from Clopidogrel to New Oral Antiplatelet Agents during Percutaneous Coronary Intervention (SCOPE). It found much higher rates of MACE, mostly driven by higher rates of myocardial infarction and transient ischemic attack/stroke in patients that de-escalated to clopidogrel [21]. However, patients de-escalating were significantly older and more often had a history of transient ischemic attacks and stroke. In addition, the de-escalation group was very small and it is unclear what the timing of events was about de-escalation.

## 4. Platelet Function Testing-Guided De-Escalation

There have been many trials investigating the use of PFT to guide antithrombotic therapy. However, almost all investigated escalation to stronger or more intensified antiplatelet regimes when high platelet reactivity was found [22,23,24]. An exception to this was the Testing Responsiveness To Platelet Inhibition On Chronic Antiplatelet Treatment For Acute Coronary Syndromes (TROPICAL-ACS) trial, which included 2610 patients and investigated de-escalation from prasugrel to clopidogrel 7 days after hospital discharge in ST-elevation myocardial infarction (STEMI) and non-ST-elevation myocardial infarction (NSTEMI) patients (all comers ACS cohort) who underwent PCI [17]. After 1 week of clopidogrel use, PFT was performed. Patients demonstrating high platelet reactivity switched back to prasugrel, while the other patients remained on clopidogrel until 12 months after myocardial infarction. Patients in the control arm were treated with prasugrel for 12 months.

The PFT guided group was non-inferior regarding the primary net clinical benefit outcome consisting of cardiovascular death, myocardial infarction, stroke or BARC grade 2 bleeding or higher (7.3% vs. 9.0% in the PFT-guided and standard treatment group respectively, P_non-inferiority_ <0.001. HR 0.81, 95% CI 0.62–1.06) [17]. The PFT guided group was also non-inferior compared to the prasugrel treated group regarding the thrombotic outcome, defined as cardiovascular death, myocardial infarction, and stroke (2.5% vs. 3.2% in the de-escalation and prasugrel group respectively, HR 0.77, 95% CI 0.48–1.21, P_non-inferiority_ = 0.012). Of note, the trial showed numerically lower bleeding events in the guided de-escalation arm (4.9% vs. 6.0% BARC 2 or higher bleeding for the de-escalation and prasugrel group, respectively, HR 0.82, 95% CI 0.59–1.13), but this difference failed to reach a level of statistical significance. A pre-specified sub-analysis of the trial assessed the impact of age clinical outcomes following PFT guided de-escalation [25]. It found that PFT guided de-escalation was associated with a significant reduction in the primary outcome in patients aged 70 and younger (HR 0.70, 95% CI 0.51–0.96, *p* = 0.03), while there was no difference in elderly patients (HR 1.17, 95% CI 0.69–2.01, *p* = 0.56). This effect was mainly driven by a reduction in bleeding events in younger patients, while it must be noted that the elderly group contained only 370 patients. The TROPICAL-ACS trial also had an important limitation; the non-inferiority margin was 30%, though in a post-hoc analysis non-inferiority for the primary endpoint was maintained with a non-inferiority margin of 10%. In addition, the PFT guided group had both numerically lower thrombotic and bleeding event rates than the standard treatment group. Further limitations included, the sole use prasugrel in the control group and not ticagrelor, excluding patients with a history of stroke, the open-label design and the loss-to-follow-up of 4% in both arms.

In TROPICAL ACS the Multiplate analyzer was used to determine platelet inhibition [17]. Correlation between different PFTs is not very good and it is therefore unsure if results can be extrapolated to other PFTs than the Multiplate [6,26]. However, the guidelines do not endorse a specific PFT and thus leave the option on what PFT to use open.

## 5. Genotype-Guided De-Escalation

The last option to guide P2Y_12_ inhibitor therapy is to use *CYP2C19* genetic testing. Similar to PFT, most trials and observational studies investigated escalation of therapy, including the recently published Tailored Antiplatelet Therapy Following PCI (TAILOR-PCI) [27,28]. In the primary analysis of this trial, which compared clopidogrel with ticagrelor in patients with loss-of-function alleles only, ticagrelor treatment led to a 34% reduction in ischemic events, though it missed statistical significance (HR 0.66, 95% CI 0.43–1.02, *p* = 0.06). This, because the trial was powered to detect a very ambitious 50% reduction in ischemic events [28]. Still, this difference is much larger than the 16% difference seen in PLATO [3].

A trial that investigated genotype-guided de-escalation was the CYP2C19 Genotype-Guided Antiplatelet Therapy in STEMI Patients–Patient Outcome after Primary PCI (POPular Genetics) trial. It investigated de-escalation from potent P2Y_12_ inhibitors to clopidogrel within 1 to 3 days after primary PCI in 2488 patients with ST-elevation myocardial infarction [18]. In patients randomized to the genotype-guided strategy the presence of the CYP2C19*2 and *3 allele was determined as soon as possible after primary PCI. Non-carriers of these alleles were de-escalated to clopidogrel, while carriers remained on potent platelet inhibitors. Median time from primary PCI to de-escalation was approximately 1.5 days. Patients in the control group received standard treatment with either ticagrelor or prasugrel for 12 months. The trial found that the genotype-guided group was non-inferior to the standard-treatment group regarding net clinical benefit, defined as all-cause death, myocardial infarction, definite stent thrombosis, stroke, and PLATO major bleeding (5.1% vs. 5.9% for genotype-guided and standard treatment respectively, P_non-inferiority_ < 0.001, HR 0.87, 95% CI 0.62–1.21). It was also non-inferior regarding the thrombotic outcome, defined as cardiovascular death, myocardial infarction, definite stent thrombosis, and stroke (2.7% vs. 3.3% for genotype-guided and standard treatment respectively, HR 0.83, 95% CI 0.53–1.31). Furthermore it found that a genotype-guided strategy was superior in reducing combined PLATO major and minor bleedings (9.8% vs. 12.5% for genotype-guided and standard treatment respectively, HR 0.78, 95% CI 0.61–0.98), which was mainly driven by a reduction in PLATO minor bleedings. Therefore, the genotype-guided therapy proved to be beneficial by reducing bleeding events, while not increasing thrombotic events. An important limitation of the POPular Genetics trial was the much lower than anticipated event rate. Since the non-inferiority margin was fixed (2%), the relative margin was much greater than expected. However, similar to the PFT guided group in the TROPICAL-ACS trial [17], patients in the genotype-guided group had numerically less thrombotic and bleeding events than patients in the standard treatment group. Other limitations included the open-label design and that more, though rare, *CYP2C19* loss-of-function alleles exist which were not tested in the trial.

## 6. Summary

**Figure 1:**
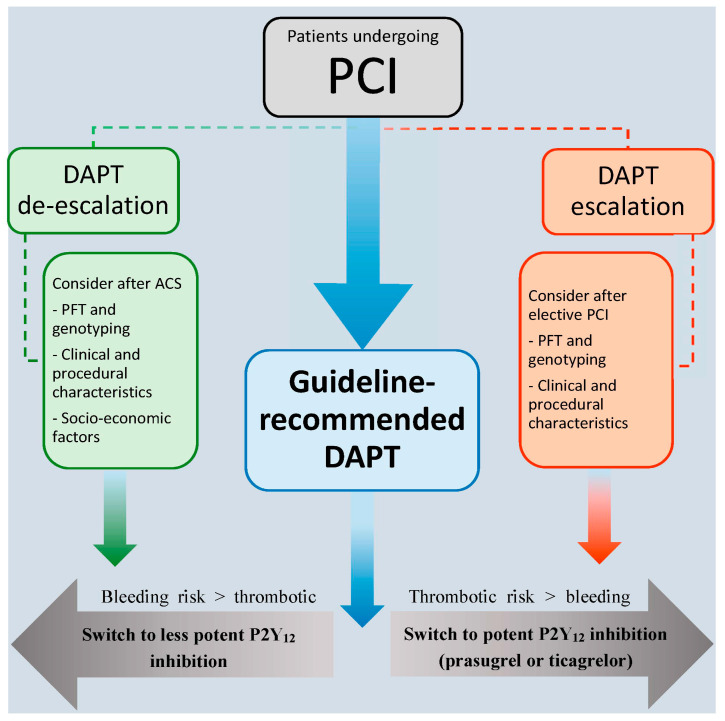
Strategies for dual antiplatelet therapy after PCI. The majority of patients undergoing percutaneous coronary intervention (PCI) should be treated with guideline recommended dual antiplatelet therapy (DAPT) (clopidogrel in elective PCI and ticagrelor or prasugrel in patients with acute coronary syndrome (ACS)). In elective PCI patients, an escalation strategy can be considered in some situations, when the thrombotic risk is higher than the bleeding risk. In ACS patients, de-escalation can be considered when the bleeding risk is higher than the thrombotic risk or for socio-economic considerations.

Based on the currently available research, the European Society of Cardiology (ESC) guidelines provide a class IIb recommendation to use unguided, PFT, or genotyping to guide antithrombotic treatment in a subgroup of ACS patients deemed unsuitable for potent platelet inhibition [29]. An expert consensus paper concerning platelet function and genetic testing to guide P2Y_12_ inhibitor treatment in PCI patients was published last year, prior to the results of the POPular Genetics trial. This consensus paper gives some suggestions as to when de-escalation in patients with myocardial infarction could be considered and when to escalate P2Y_12_ inhibitor therapy (Figure 1). In general, de-escalation should be considered in patients with a high bleeding risk (Table 2). This includes prior major bleeding, prior hemorrhagic stroke, anemia, and clinically significant bleeding on dual-antithrombotic therapy [30]. Bleeding risk scores might also be used to help decision making, though this has never been tested in a clinical trial. Furthermore, socio-economic reasons could be a factor in deciding to de-escalate therapy. If opted to de-escalate antithrombotic therapy in a patient, choosing either PFT or genetic testing both have their advantages and disadvantages (Table 3). Ultimately deciding on what strategy to use will depend on the availability of the different tests and assays, the experience and logistics in the hospital, and on the country and healthcare system the hospital is located in.

## 7. How to De-Escalate Antiplatelet Therapy

**Figure 2:**
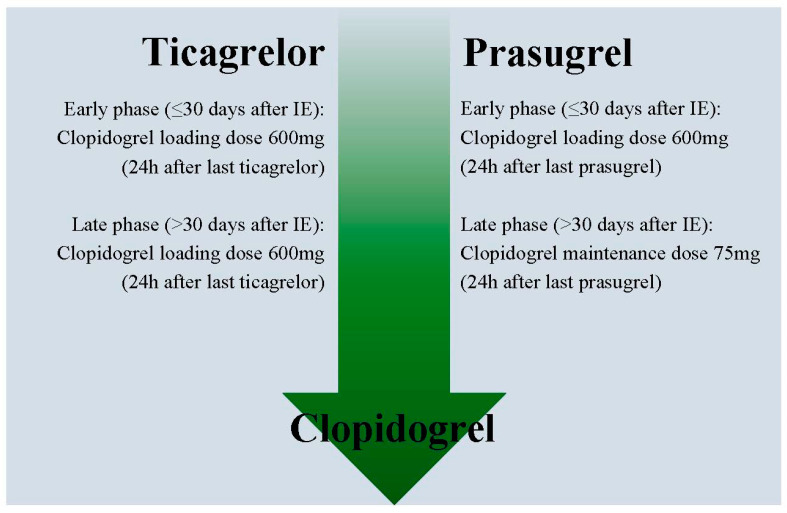
Recommended clopidogrel doses when de-escalating. When de-escalating from ticagrelor or prasugrel to clopidogrel in the early phase (≤30 days after the index event), a loading dose of 600mg should be administered 24 h after the last dose of the potent P2Y_12_ inhibitor. In the late phase (>30 days after the index event), a loading dose of 600 mg should only be administered from ticagrelor to clopidogrel, while a maintenance dose of 75 mg should be administered when de-escalating from prasugrel to clopidogrel. If de-escalating due to bleeding or bleeding concerns, a 75 mg clopidogrel dose could be considered instead of a loading dose irrespective of the phase or initial P2Y_12_ inhibitor.

After deciding to de-escalate to clopidogrel, it is possible to do this either by giving a loading dose or not. There have not been any randomized trials powered for clinical outcomes investigating this, but an expert consensus paper by Angiolillo et al. gives a recommendation based on pharmacodynamic studies. Whether or not giving a loading dose is based on the timing of de-escalation and what P2Y_12_ inhibitor is used prior to de-escalation (Figure 2) [31]. When de-escalating in the early phase (≤30 days from the index event) a 600mg loading dose of clopidogrel should be administered 24 h after the last dose of prasugrel or ticagrelor. In a sub-analysis of the POPular Genetics trial, this method seemed save with no bleeding and thrombotic events in 172 patients who switched to clopidogrel within seven days after STEMI [32]. When de-escalating from ticagrelor to clopidogrel in the late phase (>30 days from the index event) a 600 mg loading dose 24 h after the last dose of ticagrelor is recommended, while it is not recommended to use a new loading dose when switching from prasugrel to clopidogrel in the late phase. If de-escalating due to bleeding or bleeding concerns, a 75 mg clopidogrel dose could be considered instead of a loading dose irrespective of the phase or initial P2Y_12_ inhibitor [31].

## 8. Conclusions and Future Perspective

With a decline in ischemic events in the last decade, focus has shifted more and more towards preventing bleeding complications. In recent years, we have seen a growing body of evidence supporting de-escalation of antithrombotic therapy in patients with ACS who underwent PCI. Current guidelines offer the option to de-escalate in a subset of ACS patients who are not deemed suitable for potent platelet inhibitors. No new randomized data are expected in the near future. However, cost-effectiveness analyses and meta-analyses, including data from the latest randomized trials, might help expand the current recommendation. Subgroup analyses from currently published trials have not consistently identified patient groups that might benefit more from de-escalation. Therefore, for as long as guidelines do not give a higher recommendation to use de-escalation in a broader population, it will be up to the clinician to assess whether a patient could benefit or not.

## Figures and Tables

**Table 1:** Major randomized clinical trials investigating de-escalation of P2Y_12_ inhibitor treatment in patients with ACS.

Table Header	TOPIC	TROPICAL-ACS	POPular Genetics
Study Size	*n* = 646	*n* = 2610	*n* = 2488
Population	ACS + PCI (40% STEMI)	(N)STEMI + PCI (55% STEMI)	STEMI + primary PCI (100% STEMI)
Timing of De-Escalation	1 Month After ACS	7 Days After Discharge	1–3 Days After Primary PCI
Method of De-Escalation	Unguided	PFT-Guided	Genotype-Guided
Study Design	Single-Center, Randomized, Open-Label Trial of Unguided De-Escalation Vs. Standard Treatment	Randomized, Open-Label, Non-Inferiority Trial Of PFT-Guided De-Escalation Vs. Standard Treatment	Randomized, Open-Label, Non-Inferiority Trial of Genotype-Guided De-Escalation Vs. Standard Treatment
Control Arm	Ticagrelor/Prasugrel for 12 Months	Prasugrel for 12 Months	Ticagrelor/Prasugrel for 12 Months
Experimental Arm	1 Month of Ticagrelor/Prasugrel Followed By 11 Months of Clopidogrel	PFT-Guided De-Escalation With 1 Week Prasugrel Followed By 1 Week Clopidogrel, Then Depending on PFT Results Clopidogrel Or Prasugrel From Day 14 To 12 Months	*CYP2C19* Genotyping Immediately After Primary PCI. Non-Carriers of loF Alleles Switched to Clopidogrel As Soon As Possible, Carriers Continued Ticagrelor/Prasugrel for 12 Months
Primary Endpoint	1-Yr Incidence of Cardiovascular Death, Unplanned Hospitalization Leading to Urgent Coronary Revascularization, Stroke or BARC ≥ 2 Bleeding	1-Yr Incidence of Cardiovascular Death, Myocardial Infarction, Stroke or BARC ≥ 2 Bleeding	1-Yr Incidence of All-Cause Death, Myocardial Infarction, Definite Stent Thrombosis, Stroke or PLATO Major Bleeding
Key Safety Endpoint	BARC ≥2 Bleeding	BARC ≥2 Bleeding	PLATO Major and Minor Bleeding
Key Findings	**Primary Net Clinical Benefit Endpoint** (13.4% In De-Escalation Vs. 26.3% In Control Group; *P* < 0.01; HR: 0.48, 95% CI: 0.34–0.68 **Thrombotic Event Rates** Of 9.3% In De-Escalation Vs. 11.5% In Control Group; *P* = 0.36 **Bleeding Event Rates** Of 4.0% In De-Escalation Vs. 14.9% In Control Group; HR 0.30, 95% CI: 0.18–0.50; *P* < 0.01	**Primary Net Clinical Benefit Endpoint** (7.3% In De-Escalation Vs. 9.0% In Control Group; P_noninf_ <0.001; HR: 0.81, 95% CI: 0.62–1.06 **Thrombotic Event Rates** Of 2.5% In De-Escalation Vs. 3.2% In Control Group; HR 0.77, 95% CI: 0.48–1.21; P_noninf_ = 0.01 **Bleeding Event Rates** Of 4.9% In De-Escalation Vs. 6.1% In Control Group; HR 0.83, 95% CI: 0.59–1.13; *P* = 0.23	**Primary Net Clinical Benefit Endpoint** (5.1% In De-Escalation Vs. 5.9% In Control Group; P_noninf_ <0.001; HR: 0.87, 95% CI: 0.62–1.21 **Thrombotic Event Rates** Of 2.7% In De-Escalation Vs. 3.3% In Control Group; HR 0.83, 95% CI: 0.53–1.31 **Bleeding Event Rates** Of 9.8% In De-Escalation Vs. 12.5% In Control Group; HR 0.78, 95% CI: 0.61–0.98; *P* = 0.04
Funding	Investigator Initiated Trial. Funded by Hôpitaux De La Timone	Investigator Initiated Trial Funded by Roche Diagnostics. Eli Lilly & Daiichi Sankyo Company Supported Prasugrel Purchase and Drug Delivery	Investigator Initiated Trial. Funded by Netherlands Organization for Health Research and Development. Spartan Bioscience Provided Genotyping Equipment for Free

ACS = acute coronary syndrome, BARC = bleeding academic research consortium, CI = confidence interval, HR = hazard ratio, noninf = non-inferiority, LoF = loss-of-function, NSTEMI = non-ST-elevation myocardial infarction, PCI = percutaneous coronary intervention, PFT = platelet function testing, PLATO = Platelet Inhibition and Patient Outcomes, STEMI = ST-elevation myocardial infarction.

**Table 2:** Variables that could be considered for favoring de-escalation of dual antiplatelet therapy.

Prior Major Bleeding
Anemia
Clinically Significant Bleeding on Potent P2Y_12_ Inhibitors
High Bleeding Risk Defined by Bleeding Risk Scores
Socio-Economic Factors Favoring the Lower Costs of Clopidogrel
Side Effects on Prasugrel And Ticagrelor, Especially Dyspnea on Ticagrelor
Need for Triple Treatment Due to New Onset Atrial Fibrillation or Left Ventricular Thrombus After Myocardial Infarction

**Table 3:** Advantages and disadvantages of platelet function and genetic testing.

Table Header	Platelet Function Testing	Genotyping
Availability of Different Assays	Yes	Yes
Availability of Point-Of-Care Systems	Yes	Yes
Inter-Assay Variability	Yes	No
Variability of Results Over Time	Yes	No
Association with Thrombotic Events	Yes	Yes
Association with Bleeding Events	Yes	Yes
Availability of Clinical Trial Data on Guided Therapy	Yes	Yes
Feasibility in Clinical Practice	Yes	Yes
Results Influenced by Extra Patient Factors	Yes	No
Direct Measure of Response to Therapy	Yes	No
Assessment of Influence of Both Genetic and Non-Genetic Factors on Platelet Function	Yes	No
Need to Be Performed While on Treatment	Yes	No
Modified and Adapted with Permission from Sibbing Et Al. [30]		
